# “Biology and Medicine”: A Section of *Nanomaterials* Addressing Interactions of Nanomaterials with All Forms of Life

**DOI:** 10.3390/nano11092294

**Published:** 2021-09-03

**Authors:** Eleonore Fröhlich

**Affiliations:** Center for Medical Research, Medical University of Graz, Stiftingtalstr. 24, 8010 Graz, Austria; eleonore.froehlich@medunigraz.at

“Biology and Medicine” is a Section of *Nanomaterials* publishing original articles, reviews, and commentaries on any aspect related to the interaction of nanomaterials with biology. Nanomaterials have raised interest because their size-dependent physical and chemical properties improve the quality of various commercial products. Already in the late 1990s, concepts for the use of nanomaterials for medical purposes were developed [1]. One idea was to construct nanorobots to destroy caries-causing bacteria and remove blemishes on infected teeth. Although many of the planned nanoconstructs could not be realized, research on medical applications of nanomaterials continued. In 2004, the term “nanomedicine”, aiming to improve diagnosis and treatment in medicine, was defined by the European Science Foundation and in 2006 by the United States National Institutes of Health. Concerns about the potentially harmful effects of nanomaterials were stimulated by studies reporting an increased morbidity and hospitalization rate in regions with high concentrations of ultrafine particles in the air. “Nanotoxicology” summarizes the adverse effects of nanoparticle exposure [2]. Nanosafety combines research on toxicology, ecotoxicology, exposure assessment, mechanisms of interaction, risk assessment and standardization of production, characterization, and biological assessment.

Exposure to nanomaterials may occur accidentally (workplace, air pollution, release from products, waste, etc.) or intendedly (treatment, healthcare, cosmetics, etc.). There is a broad range of relevant materials (inorganic, organic, or biologic), potential exposure routes (parenteral, dermal, inhalation, oral, etc. [3]), exposed organisms (bacteria, plants, mammals, etc. [4]), and duration and concentration of the exposure. Exposure doses of nanomaterials are usually more difficult to determine than for conventional molecules, and this complicates the selection of the relevant concentration range for the testing. Further, the effect of particle agglomeration and aggregation on pharmacokinetics and pharmacodynamics has to be considered. Nanoparticle parameters, such as size, shape, chemical composition, surface structure and charge, solubility, and agglomeration and aggregation have crucial effects on organisms, organs, cells, and biomolecules. The development of new analytical methods, isolation and synthesis protocols, biological models, and prediction programs improved the understanding of the biological effects of nanomaterials. Nevertheless, there are still many questions regarding the long-term effects of nanomaterials unanswered, tissue-specific delivery needs to be improved, and the performance of multifunctional nanomaterials should be optimized. Administration of realistic doses (no overload) in the testing, consideration of interference with the assay systems, use of reference materials, absence of biological contamination/sterility of the nanomaterials, and standardized dispersion protocols are important to obtain meaningful results [5]. Standardization of nanomaterial production, characterization, and testing is the indispensable basis for reliable evaluation and comparison of nanomaterials.

The aim of the Section “Biology and Medicine” of *Nanomaterials* is to obtain further insight into the interplay of nanomaterials and biological systems and to identify efficient non-toxic nanomaterials for use in the food industry, healthcare, and medicine. Nanomaterials may be organic, inorganic, hybrid, protein-, peptide-, nucleic acid-, and lipid-based, or self-assembled biomimetic nanomaterials. Studies on the change of nanomaterial properties upon contact with physiological fluids or other biological materials, novel biological applications for nanomaterials, and the assessment of nanomaterials in physiologically relevant models are within the scope of this Section.

In summary, research interest in this Section includes the whole variety of biological interactions of nanomaterials, illustrated in Figure 1. Several key topics are listed: interaction with physiological fluids and biological matrices, development of new nanomaterials for treatment and diagnosis in animals and plants, applications of nanomaterials for biological analyses, mechanisms of toxic action, improvements for cell and tissue culture and for analysis tools using nanomaterials and information on the life cycle of nanomaterials.

## Figures and Tables

**Figure 1:**
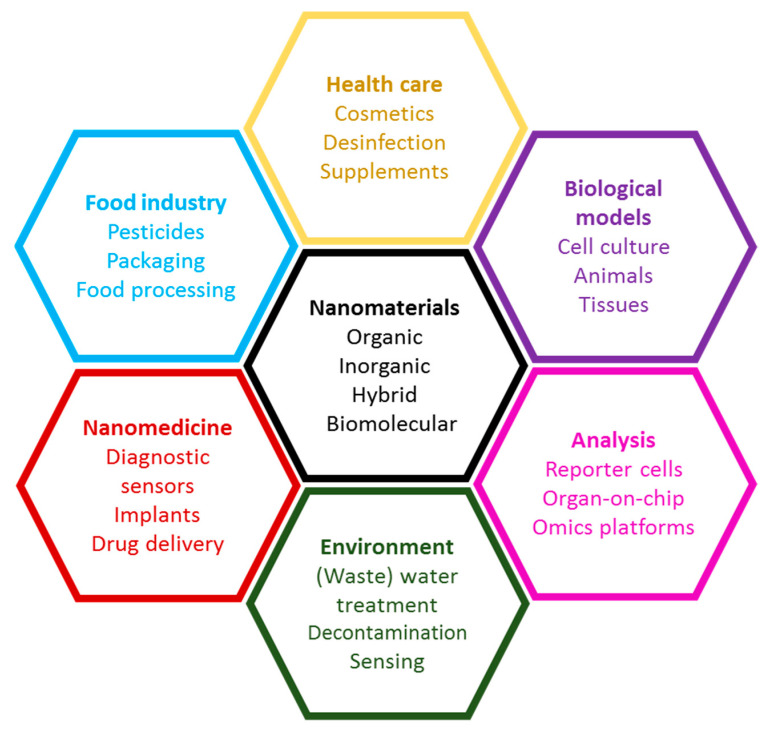
Interactions of nanomaterials with biological systems.
